# First-principles study of a penta-PdO_2_ monolayer: a 2D material for selective detection of toxic gases

**DOI:** 10.1039/d6ra04803j

**Published:** 2026-08-24

**Authors:** Methila Akter, Abu Talha, Nuzhat Nawshin, Mohammad Tanvir Ahmed, Debashis Roy, Abdullah Al Roman

**Affiliations:** a Condensed Matter Physics (CMP) Lab, Department of Physics, Jashore University of Science and Technology Jashore 7408 Bangladesh aaroman313@gmail.com

## Abstract

The development of efficient and selective gas-sensing materials is crucial for monitoring toxic atmospheric pollutants and improving environmental safety. In this study, a novel penta-PdO_2_ monolayer is proposed, and its structural, electronic, optical, and gas-sensing properties are systematically investigated using density functional theory, DFT, calculations. The calculated cohesive energy and phonon dispersion spectra confirm the energetic and dynamical stability of the monolayer, indicating its suitability for practical applications. Pristine penta-PdO_2_ exhibits semiconducting behavior with an indirect band gap of 0.855 eV. The adsorption characteristics of CO, H_2_S, NH_3_, and NO_2_ gases on the penta-PdO_2_ surface were analyzed through adsorption energy, charge transfer, electronic structure, and optical response. The results reveal that CO, H_2_S, and NH_3_ interact weakly with the surface through physisorption, causing only minor changes in the electronic properties. In contrast, NO_2_ exhibits strong chemisorption with a large adsorption energy of −0.96 eV and significant charge transfer, indicating strong interaction with the monolayer. NO_2_ adsorption substantially reduces the band gap and induces spin-polarized magnetic behavior, reflecting pronounced electronic coupling between the gas molecule and the surface. Density of states, electron density difference, and electron localization analyses further confirm the strong orbital hybridization associated with NO_2_ adsorption. Furthermore, NO_2_ significantly enhances electrical conductivity and modulates the work function, resulting in a remarkably high sensing response. Recovery time analysis also demonstrates selective retention behavior for NO_2_. These findings highlight penta-PdO_2_ as a promising two-dimensional material for selective NO_2_ adsorption and efficient sensing of CO, H_2_S, and NH_3_ gases.

## Abstract 2

The development of efficient and selective gas-sensing materials is crucial for monitoring toxic atmospheric pollutants and improving environmental safety.

## Graphical abstract

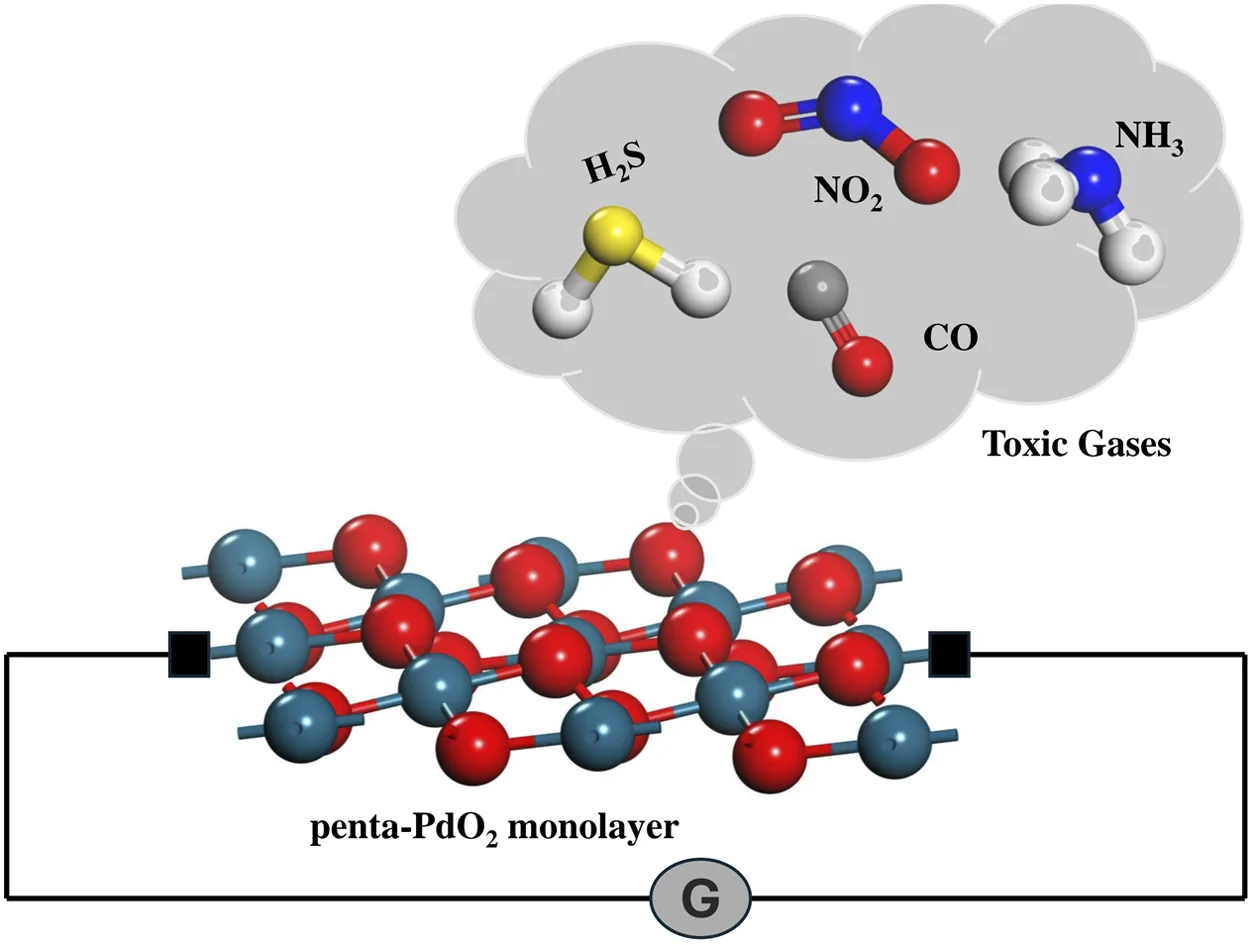

## 1. Introduction

Human activities such as fossil fuel combustion, industrial operations, agricultural practices, and waste management have increasingly degraded ambient air quality, leading to elevated levels of toxic atmospheric pollutants. These pollutants pose significant risks to ecosystems and human health, creating a need for continuous monitoring and mitigation strategies. Among the hazardous gases commonly emitted from these sources are carbon monoxide, (CO), nitrogen dioxide, (NO_2_), ammonia, (NH_3_), and hydrogen sulfide, (H_2_S), which originate from transportation, industrial processes, agriculture, and organic matter decomposition.^1,2^ Carbon monoxide, CO, is produced when carbon-containing fuels undergo incomplete combustion. It is difficult to detect in the environment because it has no color or odor.^3^ As a toxic air pollutant, it interferes with oxygen transport in the bloodstream, causing symptoms such as headache, dizziness, and nausea, and in severe cases, may lead to hypoxia or death.^4^ Hydrogen sulfide, H_2_S, is generated from organic matter decomposition and various industrial processes and is hazardous due to its combined toxicity and flammability. At elevated concentrations, it can induce respiratory irritation and neurological effects, and in severe cases, may result in death through inhibition of cellular respiration.^5^ Ammonia, NH_3_, is a toxic gas that can cause lung injury, oxidative stress, and disruption of respiratory function.^6^ In addition to its hazardous effects, NH_3_ is widely used in chemical industries and refrigeration systems as an alternative to chlorofluorocarbons, although its high volatility and toxicity pose significant risks to human health.^7^ It also plays a vital role in chemical industries for material synthesis and has recently gained attention as a promising energy carrier, while serving as an effective reducing agent in selective catalytic reduction, SCR, systems for mitigating NO_*X*_ emissions from diesel engines.^8–11^ Nitrogen dioxide, (NO_2_), a major air pollutant primarily emitted from vehicle exhaust, industrial activities, and combustion processes, is associated with chronic respiratory damage, cardiovascular and systemic diseases, and increased mortality.^12^ Beyond its adverse health effects, NO_2_ plays a significant role in photochemical smog and ozone formation and contributes to acid rain, highlighting the importance of continuous monitoring and detection.^13,14^

Monitoring hazardous gases is essential because of their negative effects on human health and the environment. This concern has encouraged the development of gas sensors with improved sensitivity and selectivity for more reliable air quality control and exposure evaluation.^15^ Two-dimensional, 2D, materials have received significant attention for toxic gas detection due to their high surface-to-volume ratio, which enables strong interaction between gas molecules and the surface, often accompanied by measurable charge transfer upon adsorption.^16–19^ Graphene is a well-known 2D material that has been extensively investigated for detecting gas molecules. Its excellent electrical behavior, including high carrier mobility and low electronic noise, supports sensitive electrical response when gases interact with its surface.^20–22^ Although sensors based on graphene can operate at room temperature, their practical sensing performance is often limited. This limitation mainly arises from the weak interaction between gas molecules and the graphene surface, along with its zero-band gap nature, which restricts effective modulation of the electronic signal. As a result, reduced sensitivity, limited selectivity, and slower response behavior are typically observed.^23,24^ Transition metal oxides, TMOs, and transition metal dichalcogenides, TMDs, have emerged as attractive candidates for room-temperature gas sensing because of their high surface-to-volume ratio, tunable electronic characteristics, intrinsic band gaps, semiconducting nature, mechanical stability, and optical transparency. These properties promote effective gas adsorption and strong modulation of the electronic response.^25–28^ A crucial requirement for effective sensors is that the target gases can bind appropriately with the sensing material. Depending on the material, these interactions may be weak, as in physisorption, or strong, as in chemisorption. For optimal sensor performance, gas–surface interactions should be of intermediate strength, ensuring sufficient adsorption for detection while still allowing rapid desorption and recovery.^29–31^ Inspired by the honeycomb lattice of graphene, Zhang *et al.* proposed a novel theoretical carbon allotrope with a pentagonal lattice, referred to as penta-graphene.^32^ This discovery has prompted the investigation of novel materials within this class. The pentagonal lattice induces a distinctive wrinkled morphology and high surface area, which facilitate tunable electronic properties, making these materials promising for high-performance gas sensing. Using first-principles calculations, Qin *et al.* analyzed the gas adsorption behavior of monolayer penta-graphene. The results show that H_2_S and NO interact strongly with the surface through chemical adsorption, whereas H_2_O, NH_3_, and SO_2_ exhibit comparatively weaker interactions. CO, on the other hand, shows only weak adsorption. These differences are also reflected in charge transfer and changes in the electronic structure of the system.^33^ In a study on Fe-doped penta-graphene, Zhang *et al.* examined the adsorption behavior of CO and CO_2_ using first-principles calculations. Their results indicate that CO interacts more strongly with the surface compared to CO_2_. The Fe dopant, introduced at sp^2^ carbon sites, was found to promote chemical bonding, along with noticeable charge transfer and modification of the electronic structure.^34^

Beyond carbon-based pentagonal lattices, pentagonal motifs have also been explored in transition metal compounds, where they exhibit interesting structural stability and electronic properties. Among these systems, PdS_2_ monolayer has attracted considerable attention due to its distinctive penta-structured framework. In this structure, Pd atoms are arranged in a planar coordination environment, while S atoms are linked through covalent bonds, forming a distorted pentagonal network similar to the bulk phase. This configuration stabilizes the monolayer and results in semiconducting behavior with an indirect band gap of approximately 1.6 eV. It also shows favorable carrier transport properties, making it relevant for electronic and sensing applications.^35^ Extending this class of materials, PdSe_2_ monolayer has also attracted attention due to its potential in gas-sensing applications. Ma *et al.* performed a first-principles study on monolayer PdSe_2_ to examine its gas adsorption behavior. The results offer insight into the interaction of different gas molecules with the surface at the electronic level. The material adsorbed NH_3_, N_2_O, NO, and NO_2_, with NO_2_ and NO showing the strongest adsorption, causing significant charge transfer, band gap reduction, and the emergence of magnetic moments. These results demonstrate high sensitivity and excellent selectivity toward nitrogen-containing gases.^36^ Correa *et al.* further reported that penta-PdSe_2_ and penta-PdPSe adsorb H_2_, CO, and NO molecules, where NO adsorption induces a change from semiconducting to metallic behavior, indicating their potential for high-performance gas sensing applications.^37^ Raval *et al.* demonstrated that penta-PdAs_2_ exhibits strong and selective physisorption toward H_2_S compared to HF and H_2_, accompanied by noticeable charge transfer and electronic modulation, highlighting the sensing potential of penta-palladium family materials.^38^ Although penta-structured Pd-based monolayers such as PdS_2_ and PdSe_2_ have demonstrated distinctive pentagonal frameworks with promising electronic and gas-sensing properties, their scope remains limited to chalcogenide- and pnictogen-based compounds. Notably, oxygen-containing penta-materials have not been investigated, leaving a critical gap in understanding how substitution of chalcogen and pnictogen atoms with oxygen modifies the fundamental Pd–X bonding characteristics within pentagonal frameworks. In contrast to PdS_2_ and PdSe_2_, where Pd–chalcogen interactions govern the electronic structure, substitution of highly electronegative oxygen is expected to strengthen Pd–O hybridization, leading to enhanced charge redistribution and significant modulation of the electronic states near the Fermi level. These effects are expected to significantly alter adsorption energy and charge-transfer behavior, thereby distinguishing penta-PdO_2_ from previously reported penta-palladium systems. Motivated by this, the present study proposes a novel penta-PdO_2_ monolayer and systematically investigates its structural, electronic, and optical properties, along with its gas-sensing behavior toward CO, H_2_S, NH_3_, and NO_2_ molecules using first-principles calculations.

## 2. Computational details

All calculations were performed using CASTEP within density functional theory, DFT, to investigate the structural, electronic, and optical properties of the penta-PdO_2_ monolayer upon gas adsorption. The exchange–correlation interactions were described using the generalized gradient approximation, GGA, in the Perdew–Burke–Ernzerhof, PBE, form.^39^ Since gas adsorption on two-dimensional monolayers is strongly influenced by long-range van der Waals interactions, Grimme's DFT-D2 semi-empirical correction was employed throughout the calculations to accurately describe the adsorption behavior between the gas molecules and the penta-PdO_2_ surface.^40,41^

The pristine penta-PdO_2_ structure was fully optimized following plane-wave cutoff energy convergence tests in the range of 600 to 700 eV, with 700 eV selected as the optimal value ensuring total energy convergence and computational reliability. Geometry optimization of the primitive cell was performed using a Monkhorst–Pack *k*-point mesh of 6 × 6 × 1. For adsorption calculations, a 2 × 2 × 1 supercell was constructed, and a vacuum layer of 20 Å was introduced along the *z*-direction to eliminate interlayer interactions between periodic images. Electronic structure and optical properties were evaluated using a denser 3 × 3 × 1 *k*-point grid within the Monkhorst–Pack scheme, while retaining the same plane-wave cutoff energy as used in structural optimization.^42^ Spin-polarized calculations were performed for pristine penta-PdO_2_ and the NO_2_-adsorbed system due to the presence of an unpaired electron and associated magnetic character. In contrast, non-spin-polarized calculations were applied to CO, H_2_S, and NH_3_ adsorbed systems owing to their closed-shell electronic configurations.^36^ All structures were optimized using stringent convergence criteria to ensure high numerical accuracy. Convergence was achieved when the total energy change per atom was less than 1 × 10^−5^ eV, atomic forces were below 0.03 eV Å^−1^, ionic displacement was less than 0.001 Å, and stress was within 0.05 GPa.^43^ To identify the most stable adsorption geometries, the adsorption locator tool was employed to determine the minimum-energy configurations of gas molecules on the penta-PdO_2_ surface.^44^ To evaluate the thermal stability of the penta-PdO_2_ monolayer, molecular dynamics simulations were performed using the Forcite module under the microcanonical, NVE, ensemble at 300 K for 10 ps.^45^ The strength of adsorption can be quantitatively evaluated through the adsorption energy, which for the gas molecules on PdO_2_ is defined as follows:^46^

1 *E*_ads_ = *E*_PdO_2_+gas_ − *E*_PdO_2__ − *E*_gas_

where, *E*_PdO_2_+gas_, *E*_PdO_2__, and *E*_gas_ denote the total energies of the gas-adsorbed system, pristine nanosheet, and isolated gas molecule, respectively. The noncovalent interaction, NCI, analysis was performed using Multiwfn 3.8, with wave function files obtained from the optimized geometries as input, and the resulting interactions were visualized using Visual Molecular Dynamics, VMD, software.^47–49^

## 3. Results and discussion

### 3.1 Structural properties and stability analysis

**Fig. 1:**
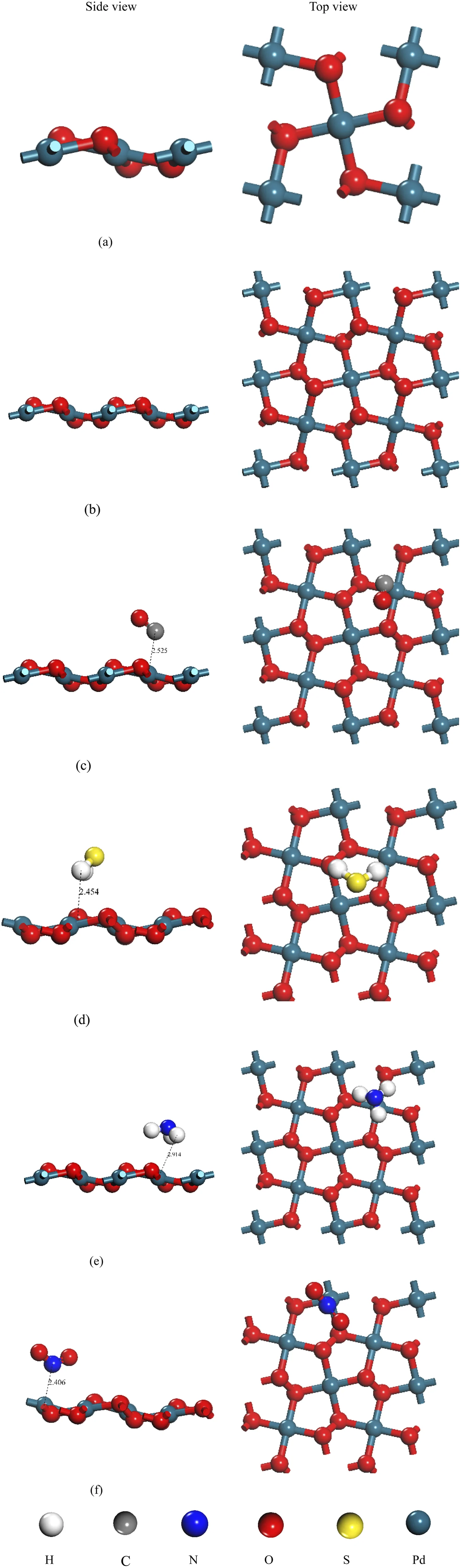
Optimized structures, side and top views, of penta-PdO_2_ and gas adsorbed penta-PdO_2_. (a) Unit cell (PdO_2_). (b) Supercell (PdO_2_). (c) CO + PdO_2_. (d) H_2_S + PdO_2_. (e) NH_3_ + PdO_2_. (f) NO_2_ + PdO_2_.

A geometrically stable structure is essential for the reliable evaluation of the functional properties of the material.^50^ The penta-PdO_2_ structure was constructed with 24 atoms, comprising 8 Pd and 16 O atoms. Each Pd atom adopts a square-planar coordination with four neighboring O atoms, while each O atom forms two Pd–O bonds and one O–O bond. This bonding arrangement produces a wrinkled pentagonal unit consisting of two Pd atoms and three O atoms, forming the distinctive pentagonal lattice of the monolayer. The optimized lattice constants of the penta-PdO_2_ primitive cell are *a* = 4.78 Å and *b* = 4.76 Å. Fig. 1 presents the relaxed geometries of pristine and gas-adsorbed penta-PdO_2_ in both top and side views, allowing a direct comparison between the two configurations. The corresponding bond length variations are summarized in Table 1. After structural optimization, the penta-PdO_2_ monolayer attains Pd–O and O–O bond lengths of 2.046 Å and 1.492 Å, respectively. For the isolated gas molecules, the optimized bond lengths are calculated as 1.153 Å for CO, 1.350 Å for H_2_S, 1.029 Å for NH_3_, and 1.230 Å for NO_2_, corresponding to the C≡O, H–S, N–H, and N–O bonds. These results are in good agreement with previous studies.^43,51^

**Table 1:** Comparison of bond lengths, Å, of penta-PdO_2_ and gas molecules before adsorption, *B*_ad_, and after adsorption

Bond type	PdO_2_	PdO_2_ + CO	PdO_2_ + H_2_S	PdO_2_ + NH_3_	PdO_2_ + NO_2_
Pd–O	2.043	2.052	2.040	2.044	2.042
O–O	1.492	1.490	1.497	1.498	1.485
C≡O	—	1.159, 1.153 (*B*_ad_)	—	—	—
H–S	—	—	1.352, 1.350 (*B*_ad_)	—	—
N–H	—	—	—	1.045, 1.029 (*B*_ad_)	—
N–O	—	—	—	—	1.240, 1.230 (*B*_ad_)

Upon adsorption of gas molecules on the penta-PdO_2_ monolayer, only slight structural variations are observed in both the substrate and the adsorbates. The Pd–O and O–O bond lengths of the monolayer exhibit minor changes, while the internal bonds of the gas molecules, C≡O, H–S, N–H, and N–O, also show subtle adjustments.

The cohesive energy, (*E*_coh_), which quantifies the intrinsic binding strength of the monolayer, is calculated using the following equation:^43^2
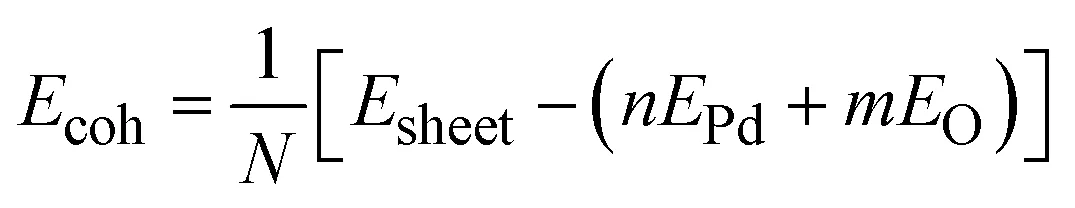
Here, *E*_sheet_ denotes the total energy of the penta-PdO_2_ monolayer, while *E*_Pd_ and *E*_O_ correspond to the energies of isolated Pd and O atoms, respectively. The parameters *n* and *m* define the number of Pd and O atoms in the sheet, and *N* represents the total number of atoms in the sheet. A cohesive energy of −4.73 eV per atom is obtained for penta-PdO_2_, indicating its structural stability due to the negative value.

**Fig. 2:**
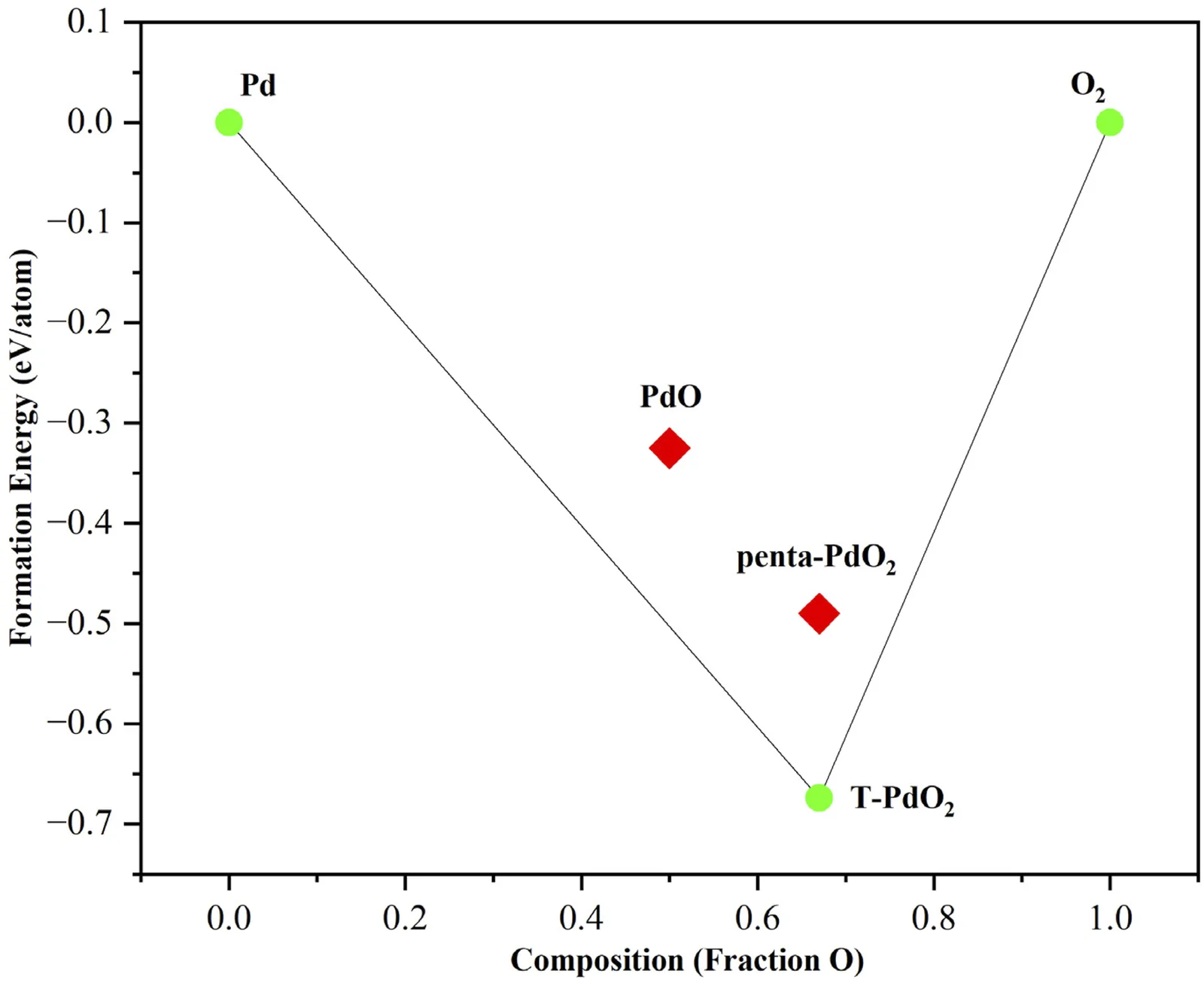
Binary phase diagram of the Pd–O system constructed from the calculated formation energies, showing the convex hull of thermodynamically stable phases and the position of the proposed penta-PdO_2_ structure relative to the hull.

To further evaluate the thermodynamic stability of the proposed penta-PdO_2_ monolayer, the convex-hull phase diagram of the two-dimensional Pd–O system was constructed based on the calculated formation energies of competing Pd–O phases, including the previously reported T-PdO_2_ and PdO monolayers,^52,53^ as shown in Fig. 2. The convex hull defines the thermodynamically stable phases at each composition, while structures located above the hull are considered metastable. As shown in Fig. 2, T-PdO_2_ lies on the convex hull, indicating that it is the thermodynamic ground state at the PdO_2_ composition. In contrast, the proposed penta-PdO_2_ monolayer lies approximately 0.18–0.20 eV per atom above the convex hull, indicating that it is a metastable phase rather than the thermodynamic ground state. Although the positive energy above the hull indicates a thermodynamic driving force for decomposition into competing Pd–O phases, the negative formation energy, high cohesive energy, absence of imaginary phonon modes, and preservation of the structural framework during finite-temperature molecular-dynamics simulations demonstrate its energetic, dynamical, and thermal stability as an isolated monolayer. Therefore, penta-PdO_2_ may be considered a metastable but structurally robust candidate, whose experimental realization could potentially depend on kinetically controlled synthesis or substrate-assisted stabilization.

### 3.2 Phonon dispersion and dynamical stability

Dynamical stability was confirmed *via* phonon dispersion analysis. A crystal structure is considered dynamically stable when all phonon modes exhibit real, positive frequencies across the entire Brillouin zone. Conversely, phonon modes with imaginary, negative, frequencies indicate that atomic displacements lead to non-restoring forces, resulting in an unstable lattice configuration and confirming dynamical instability.^54^ The phonon band structure of the system was computed using density functional theory within the GGA framework, employing the PBE exchange–correlation functional.

**Fig. 3:**
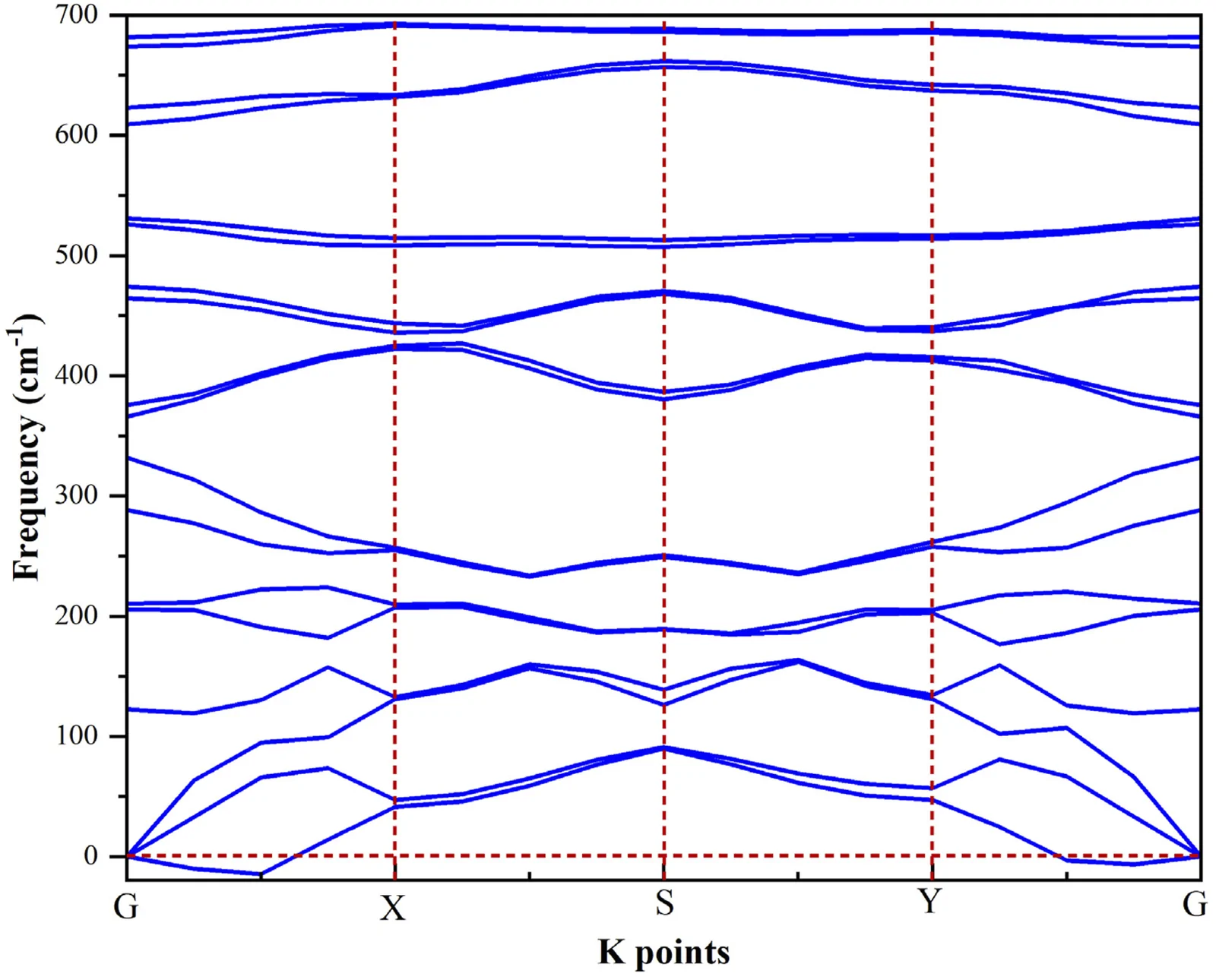
Phonon dispersion curve of penta-PdO_2_.

Fig. 3 presents the phonon dispersion spectrum of penta-PdO_2_ calculated along the high-symmetry path G–X–S–Y–G, with vibrational frequencies extending up to 700 cm^−1^. Owing to the presence of six atoms in the primitive unit cell, the system exhibits 18 phonon branches, (3N modes), including three acoustic modes consisting of one longitudinal acoustic, (LA), one transverse acoustic, (TA), and one out-of-plane flexural acoustic, ZA, branch.^55^ Importantly, no significant imaginary phonon frequencies are observed throughout the Brillouin zone, confirming the dynamical stability of the optimized structure. Only a very small negative frequency appears near the Γ point in the ZA branch, which is commonly encountered in two-dimensional materials and is generally attributed to numerical convergence limitations associated with long-wavelength out-of-plane vibrations rather than genuine structural instability.^56–58^ Similar minor imaginary features have been widely reported for stable 2D monolayers and are known to diminish upon stricter convergence settings or improved symmetry treatment.^55,57^ Furthermore, such flexural instabilities can be effectively suppressed under practical conditions through substrate support or external constraints.^56^ It should be noted that the present phonon calculations describe the intrinsic dynamical stability of a free-standing penta-PdO_2_ monolayer. In practical gas-sensing devices, the monolayer would be supported on an appropriate substrate and integrated with metallic electrodes, where interfacial interactions may slightly influence its structural and electronic characteristics. Therefore, the phonon analysis confirms that penta-PdO_2_ possesses robust dynamical stability, supporting its feasibility for potential experimental realization and device applications.

### 3.3 Molecular dynamics

**Fig. 4:**
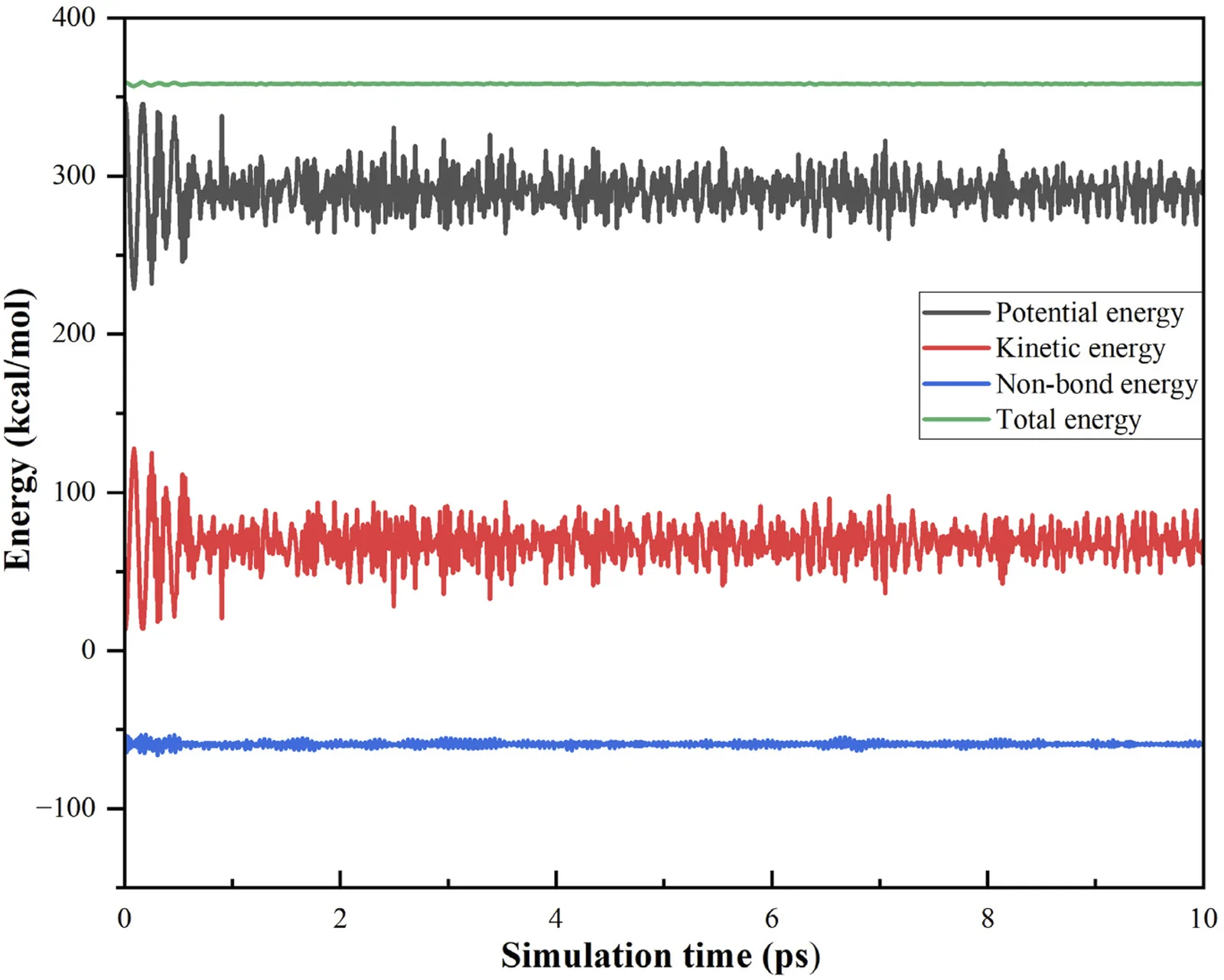
Molecular dynamics simulation of penta-PdO_2_ monolayer under NVE at 300 K.

To further evaluate the finite-temperature stability of the proposed penta-PdO_2_ monolayer, molecular dynamics, MD, simulations were performed at 300 K using the microcanonical, NVE, ensemble. The simulations were carried out for 5 ps with a time step of 1 fs to monitor the structural evolution of the system. The variations in the total, potential, kinetic, and non-bonded energies during the MD simulation are presented in Fig. 4. Throughout the simulation, the total energy remained nearly constant, exhibiting only minor fluctuations and no noticeable energy drift, indicating excellent energy conservation in the NVE ensemble. The calculated total energy fluctuation of approximately 0.064% is significantly lower than the generally accepted threshold for stable MD simulations, further confirming the reliability of the calculations. Moreover, no bond breaking, structural reconstruction, or significant lattice distortion was observed during the entire simulation. These results demonstrate that the penta-PdO_2_ monolayer retains its structural integrity under ambient conditions, confirming its excellent thermal stability and supporting its potential for future experimental realization and practical gas-sensing applications^59,60^

### 3.4 Adsorption energy

**Fig. 5:**
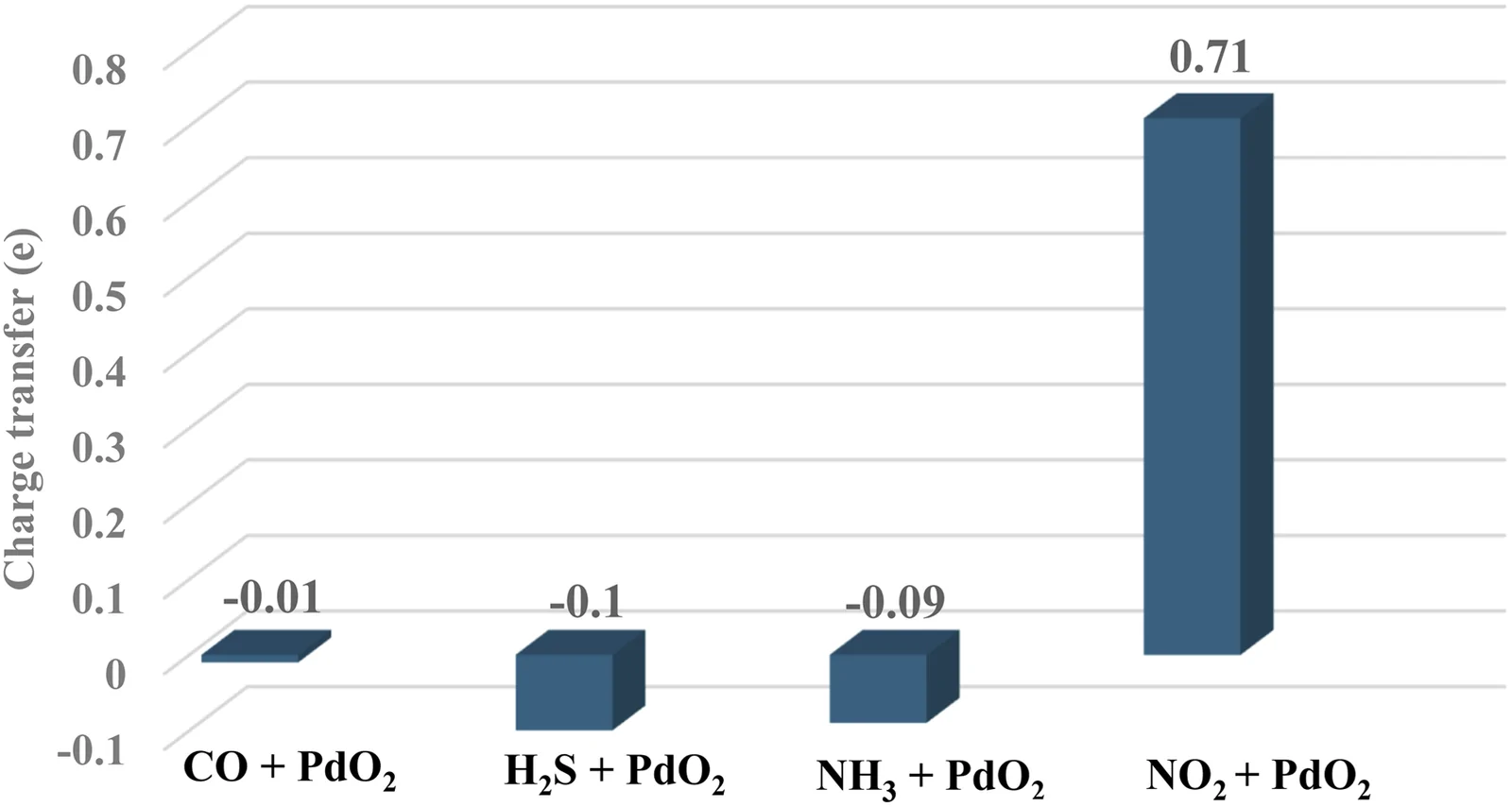
Charge transfer between the penta-PdO_2_ substrate and adsorbed gas molecules.

Adsorption energy is used as a quantitative measure to describe the interaction between gas molecules and the sensing surface, allowing assessment of binding strength and sensor performance.^61^ The gas sensing performance of pristine penta-PdO_2_ is assessed through a systematic investigation of its interactions with CO, H_2_S, NH_3_, and NO_2_ molecules. Adsorption energies for gas molecules on the penta-PdO_2_ monolayer are evaluated using eqn (1). A negative value of *E*_ads_ corresponds to an exothermic adsorption process, indicating that the interaction between the gas molecules and the penta-PdO_2_ surface is energetically favorable.^62^ Generally, an adsorption energy with an absolute magnitude greater than about −0.5 eV is considered indicative of chemisorption.^63^ Among the studied gases, NO_2_ exhibits the strongest interaction with the penta-PdO_2_ monolayer, with an adsorption energy of −0.96 eV. NO_2_ adsorption induces a significant charge transfer of 0.71 e from the molecule to the penta-PdO_2_ surface, indicating strong electronic interaction between the adsorbate and the substrate. The corresponding charge transfer distribution is illustrated in Fig. 5 confirming strong chemisorption, whereas CO, H_2_S, and NH_3_ exhibit only small charge transfer, indicating weak physisorption interactions. Charge transfer plays an important role in the sensing behavior of two-dimensional materials, as gas adsorption induces electron redistribution between the adsorbate and the penta-PdO_2_ surface, depending on the electronic nature of the gas molecules.^64^ At the most stable adsorption site, the NO_2_ molecule is located at a minimum distance of 2.406 Å from the penta-PdO_2_ surface. Compared to the free NO_2_ molecule, (N–O bond length 1.230 Å and O–N–O bond angle 133.445°), the adsorbed NO_2_ shows a slight elongation of the N–O bond to 1.240 Å and a reduction of the O–N–O angle to 128.897°. For the penta-PdO_2_ monolayer, the Pd–O and O–O bond lengths change slightly from 2.043 and 1.492 Å to 2.042 and 1.485 Å, respectively, indicating minimal structural distortion of the penta-PdO_2_. CO, H_2_S, and NH_3_ adsorb at minimum distances of 2.525 Å, 2.454 Å, and 2.914 Å from the penta-PdO_2_ surface, respectively, accompanied by electron transfer to the monolayer, indicating varying degrees of charge redistribution. By comparison, the adsorption of CO, H_2_S, and NH_3_ is significantly weaker, being approximately 0.5 eV lower than that of NO_2_, indicating that their interactions with penta-PdO_2_ occur primarily *via* physisorption.

For comparison, the reported adsorption energy of NO_2_ on penta-PdSe_2_, −0.579 eV, is notably smaller, indicating a weaker interaction strength.^36^ By contrast, NO_2_ adsorption on Pd-doped HfSe_2_ is relatively weak and corresponds to physisorption, whereas penta-PdO_2_ exhibits much stronger interactions, highlighting its superior sensing potential.^65^ Compared with NH_3_ adsorption on penta-PdSe_2_, −0.287 eV,^36^ penta-PdO_2_ exhibits stronger NH_3_ adsorption. Table 6 presents a comparative evaluation of the gas-sensing performance of various 2D nanomaterials based on key sensing parameters.

Deformation energy analysis is employed to assess the structural stability of the penta-PdO_2_ monolayer following gas adsorption. The deformation energy, *E*_def_, is obtained from the energy difference between the deformed and pristine, undeformed, configurations of the adsorbent, as given in the following equation^49^3
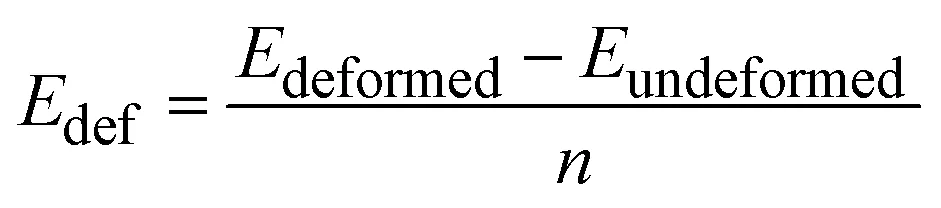
Here, *E*_undeformed_ corresponds to the total energy of the fully relaxed penta-PdO_2_ monolayer, while *E*_deformed_ refers to the total energy of the system after structural distortion induced by gas adsorption. Once the adsorbed gas molecules were removed, the energy of the deformed monolayer was recalculated to quantify the induced structural change. The calculated deformation energies for all gas molecules are very small, (∼10^−3^ eV per atom), indicating negligible structural distortion and excellent structural stability of the penta-PdO_2_ monolayer upon adsorption. Among the investigated gases, NH_3_ induces the highest deformation, followed by H_2_S and CO, whereas NO_2_ causes the lowest deformation. Although NO_2_ exhibits comparatively strong chemisorption, the corresponding low deformation energy suggests that the adsorption process is primarily dominated by charge transfer and orbital hybridization rather than significant lattice distortion, which may be attributed to the rigid Pd–O pentagonal structure and the different adsorption configuration compared with the other gas molecules. These findings demonstrate that penta-PdO_2_ preserves its structural integrity during gas adsorption, highlighting its potential as a stable and reusable material for gas-sensing applications.^66^ The calculated deformation energies for all studied gas molecules are summarized in Table 2.

**Table 2:** Represents the calculated adsorption energy, (*E*_ads_), minimum adsorption distance, (*d*_min_), recovery time, (*τ*), charge transfer, (*Q*), magnetic moment, (MM), and deformation energy, *E*_def_, induced upon gas adsorption on the penta-PdO_2_ monolayer

Structures	*E*_ads_, (eV)	*d*_min_, (Å)	MM, (*µ*_B_)	*Q*, (e)	*τ*, (s)	*E*_def_, (eV per atom)
PdO_2_ + CO	−0.413	2.525	—	−0.01	9.6 × 10^−6^	7.45 × 10^−3^
PdO_2_ + H_2_S	−0.378	2.454	—	−0.10	2.4 × 10^−6^	7.55 × 10^−3^
PdO_2_ + NH_3_	−0.448	2.914	—	−0.10	3.7 × 10^−5^	7.85 × 10^−3^
PdO_2_ + NO_2_	−0.960	2.406	0.71	0.71	1.7 × 10^4^	6.83 × 10^−3^

Furthermore, an efficient gas sensor should be reusable, which requires a short recovery time, *τ*, to allow rapid desorption of the adsorbed molecules. The recovery time, *τ*, is calculated using the following expression:^67^4
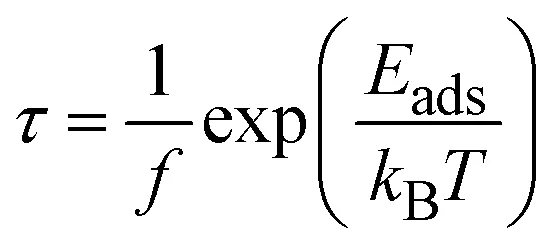
Here, *E*_ads_, *k*_B_, *T*, and *ν*_0_ represent the adsorption energy, Boltzmann constant, absolute temperature, and attempt frequency, respectively. In eqn (4), *f*, is the attempt frequency, pre-exponential factor, associated with the desorption process. Following previous theoretical studies, *f*, is taken as be 10^12^ s^−1^ corresponding to the typical vibrational frequency of adsorbed gas molecules.^68^ The calculated recovery times should be regarded as theoretical estimates because they neglect surface defects, humidity, substrate effects, and other environmental factors that may influence desorption under experimental conditions.

Recovery times were evaluated at 298 K under UV irradiation, 10^12^ s^−1^, to simulate practical sensor operating conditions and surface regeneration. As summarized in Table 2, CO, H_2_S, and NH_3_ exhibit very short recovery times in the microsecond range, indicating rapid desorption from the penta-PdO_2_ surface. Such behavior originates from their relatively weak adsorption strength and physisorption-dominated interactions, which are beneficial for reversible sensing and fast cyclic operation. Among these gases, NH_3_ shows a slightly longer recovery time due to its comparatively stronger adsorption, although it still remains suitable for practical reusable sensing applications. In contrast, NO_2_ exhibits a significantly longer recovery time, (∼1.7 × 10^4^ s), which is directly associated with its strong chemisorption and high adsorption energy on the penta-PdO_2_ surface. The strong electronic coupling and substantial charge transfer create a higher desorption barrier, slowing the intrinsic recovery process under ambient conditions. Although prolonged recovery may limit rapid cycling performance, it also reflects the exceptional selectivity and sensing stability of the monolayer toward NO_2_. Moreover, such long recovery times can be reduced experimentally through external activation methods such as thermal treatment or enhanced UV illumination, which accelerate desorption kinetics.^69,70^

Overall, the recovery time analysis indicates that penta-PdO_2_ provides a balanced sensing performance, combining rapid and reversible detection for weakly adsorbed gases with highly selective and stable NO_2_ sensing.

### 3.5 Mulliken charge analysis

Mulliken charge analysis is employed to examine the distribution of electronic charge within the system and its variation after gas adsorption, thereby enabling evaluation of the bonding characteristics between the adsorbate and the penta-PdO_2_ monolayer.^71^ Mulliken charge analysis indicates that in penta-PdO_2_, Pd atoms carry partial positive charges, +0.63 e, and O atoms partial negative charges, −0.32 e, on average. This unequal charge distribution reflects the polar covalent nature of Pd–O bonds, where electron density is preferentially shifted toward O due to its higher electronegativity, imparting a partially ionic character. The Pd and O atoms in penta-PdO_2_ retain their original charge characteristics upon adsorption of CO, H_2_S, NH_3_, and NO_2_, with Pd remaining partially positive and O partially negative, indicating that the sheet's overall polarity is largely preserved. Among the adsorbed gas molecules, hydrogen and carbon atoms carry partial positive charges, whereas oxygen, sulfur, and nitrogen atoms are partially negative, reflecting polar interactions with the surface. Specifically, in H_2_S, the sulfur atom exhibits a partial negative charge, functioning as an electron acceptor, whereas in NH_3_ and NO_2_, nitrogen atoms carry negative charges, highlighting their electron-accepting nature. Notably, the nitrogen atom in NH_3_ carries a strong negative charge, while the hydrogen atoms are partially positive, reflecting the polarization within the NH_3_ molecule and the transfer of electron density from the electron-rich nitrogen toward the PdO_2_ surface, establishing polar interactions. In contrast, upon NO_2_ adsorption on penta-PdO_2_, the nitrogen atom becomes partially positive while the oxygen atoms remain negative, reflecting the high electronegativity of oxygen and the resulting electron withdrawal from nitrogen, which reverses the donor–acceptor behavior observed in NH_3_. This redistribution of electron density directly affects the material's conductivity: electron transfer from the gas to the PdO_2_ surface, or *vice versa*, modifies the local charge density on the surface, altering the carrier concentration. As a result, the magnitude and direction of charge transfer, as quantified by Mulliken analysis, correlate with the sensing response, with stronger charge transfer generally producing a higher sensitivity toward a given gas molecule.

### 3.6 Band gap analysis

**Fig. 6:**
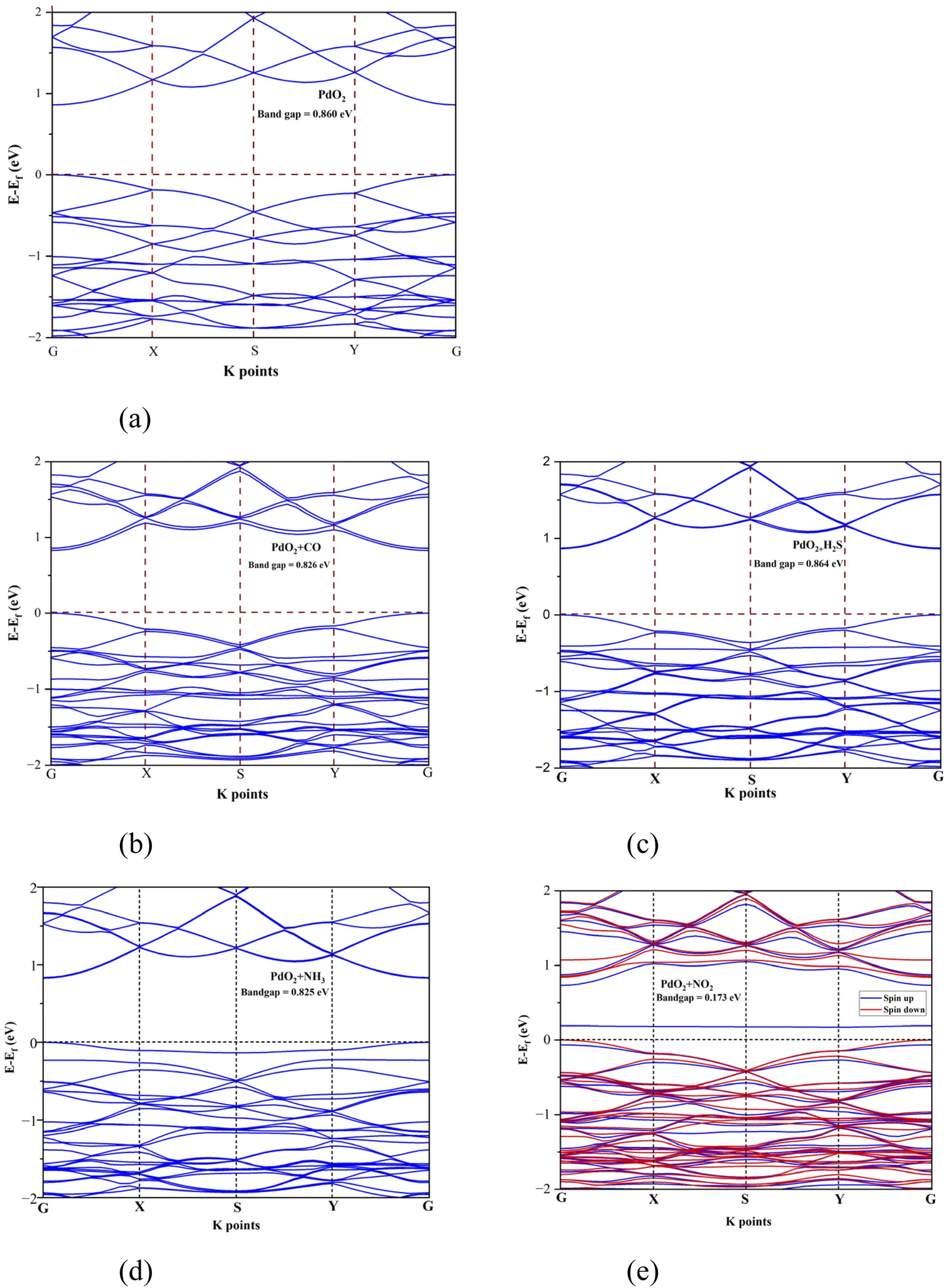
Band structures of penta-PdO_2_ and gas-adsorbed penta-PdO_2_. (a) PdO_2_. (b) CO + PdO_2_. (c) H_2_S + PdO_2_. (d) NH_3_ + PdO_2_. (e) NO_2_ + PdO_2_.

The calculated band structure of penta-PdO_2_ for both pristine and gas-interacted configurations along the G–X–S–Y–G path is shown in Fig. 6, highlighting the changes in electronic structure induced by adsorption. Spin-polarized calculations confirm that pristine penta-PdO_2_ possesses a non-magnetic semiconducting ground state with an indirect band gap of 0.855 eV. After adsorption of CO, H_2_S, and NH_3_, only minor changes are observed in the band structures of penta-PdO_2_, and the indirect semiconducting character is largely preserved. This behavior is associated with the weak coupling between the adsorbed molecules and the PdO_2_ surface, where the molecular electronic states are located far from the Fermi level.^36^

In contrast, NO_2_ adsorption significantly modifies the electronic structure. Due to its open-shell nature, spin polarization was included in the calculations. In the NO_2_-adsorbed system, pronounced spin splitting is observed, accompanied by spin-dependent impurity states near the Fermi level. These states exhibit strong hybridization with the PdO_2_ orbitals and result in a reduced band gap of 0.173 eV. While the system retains an indirect band gap, the VBM remains along X–S, and the CBM shifts toward S and S–Y in different spin channels, indicating spin-dependent narrow-gap behavior. These results demonstrate the markedly stronger interaction of NO_2_ with penta-PdO_2_ relative to other gases, highlighting its potential for selective NO_2_ sensing.

### 3.7 Density of states analysis

**Fig. 7:**
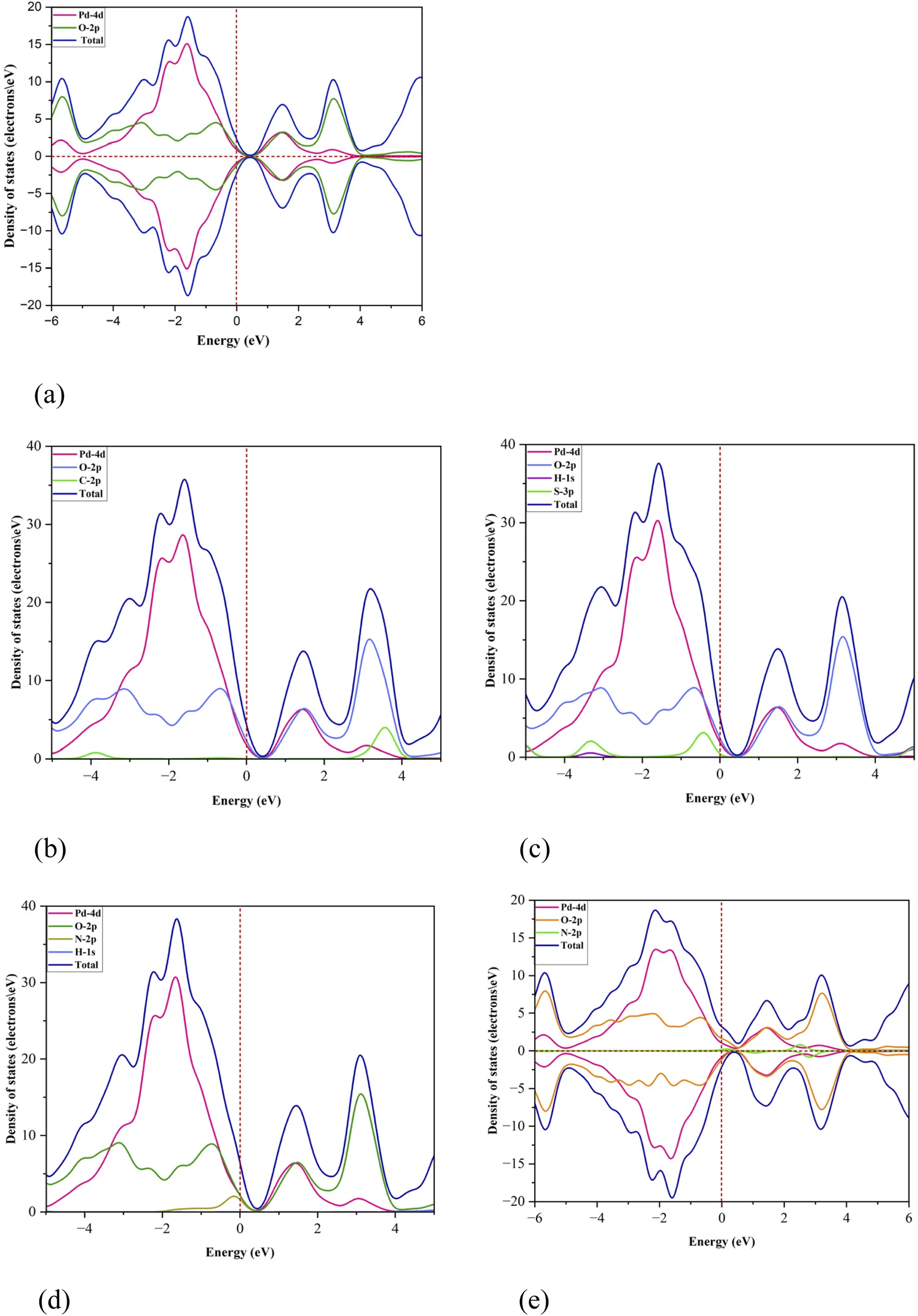
Density of states of the penta-PdO_2_ and gas-adsorbed penta-PdO_2_. (a) PdO_2_. (b) CO + PdO_2_. (c) H_2_S + PdO_2_. (d) NH_3_ + PdO_2_. (e) NO_2_ + PdO_2_.

The electronic behavior of the system is explored through total and partial density of states, DOS and PDOS, calculations. Instead of relying only on band structure, the DOS provides a clearer picture of how electronic states are distributed across different energy levels. In particular, attention is given to the region near the Fermi level, (*E*_F_), since this part largely governs the electrical response of the material. If states are present in this region, electrons can move freely, indicating metallic behavior. On the other hand, the absence of states around the Fermi level reflects the presence of an energy gap, which is characteristic of semiconducting or insulating systems.^72^Fig. 7 displays the total and partial density of states, DOS and PDOS, of the penta-PdO_2_ monolayer in pristine and gas-adsorbed states. For pristine penta-PdO_2_, no electronic states are observed at the Fermi level, confirming its intrinsic semiconducting nature. The spin-up and spin-down channels are completely symmetric, indicating a nonmagnetic ground state with zero net magnetic moment.^73^ The valence band is primarily dominated by strong hybridization between Pd-4d and O-2p orbitals, particularly within the energy range from −4 to −1 eV, reflecting significant Pd–O covalent interaction. In contrast, the conduction band is mainly governed by O-2p states with relatively weaker Pd-4d contributions, indicating reduced orbital coupling above the Fermi level.

Upon adsorption of CO, H_2_S, and NH_3_, the overall electronic structure of penta-PdO_2_ remains largely preserved. The states near the Fermi level continue to be dominated by Pd-4d and O-2p orbitals, whereas the molecular orbitals of the adsorbed gases contribute only weakly in this region. In the CO-adsorbed system, the C-2p states exhibit negligible overlap with the substrate orbitals, suggesting weak electronic coupling and physisorption behavior. Similarly, H_2_S adsorption introduces only minor contributions from S-3p and H-1s orbitals near EF, indicating limited perturbation of the substrate electronic structure. For NH_3_ adsorption, a slight contribution from N-2p states appears close to the Fermi level, implying relatively stronger interaction compared with CO and H_2_S; however, the semiconducting character of penta-PdO_2_ remains essentially unchanged. These results are consistent with the weak adsorption energies and small charge-transfer values obtained for these molecules. In contrast, NO_2_ adsorption induces substantial modification of the electronic structure. The spin-resolved PDOS reveals pronounced asymmetry between the spin-up and spin-down channels near the Fermi level, confirming adsorption-induced spin polarization. Significant overlap between Pd-4d orbitals and NO_2_ molecular states is observed around EF, indicating strong orbital hybridization and electronic coupling between the adsorbate and the substrate. This interaction introduces impurity states within the bandgap region, resulting in a remarkable reduction of the band gap from 0.855 eV to 0.173 eV. The broadened NO_2_-derived states further indicate enhanced electron delocalization upon adsorption.

Moreover, NO_2_ adsorption induces a magnetic moment of approximately 1.0 µB due to the strong interaction between the unpaired electron of NO_2_ and the neighboring Pd atom. The spin density remains mainly localized around the adsorption site, confirming adsorption-induced local magnetization. The strong Pd-4d–NO_2_ orbital hybridization, pronounced spin polarization, and significant band-gap reduction demonstrate substantial electronic modulation after NO_2_ adsorption.^74–76^ In contrast, CO, H_2_S, and NH_3_ interact weakly with the penta-PdO_2_ surface through limited orbital overlap, preserving the intrinsic semiconducting nature of the monolayer. Overall, the PDOS results highlight the exceptional sensitivity and selectivity of penta-PdO_2_ toward NO_2_ sensing and suggest its potential for magnetic gas-sensing applications.

### 3.8 Electron density difference analysis

**Fig. 8:**
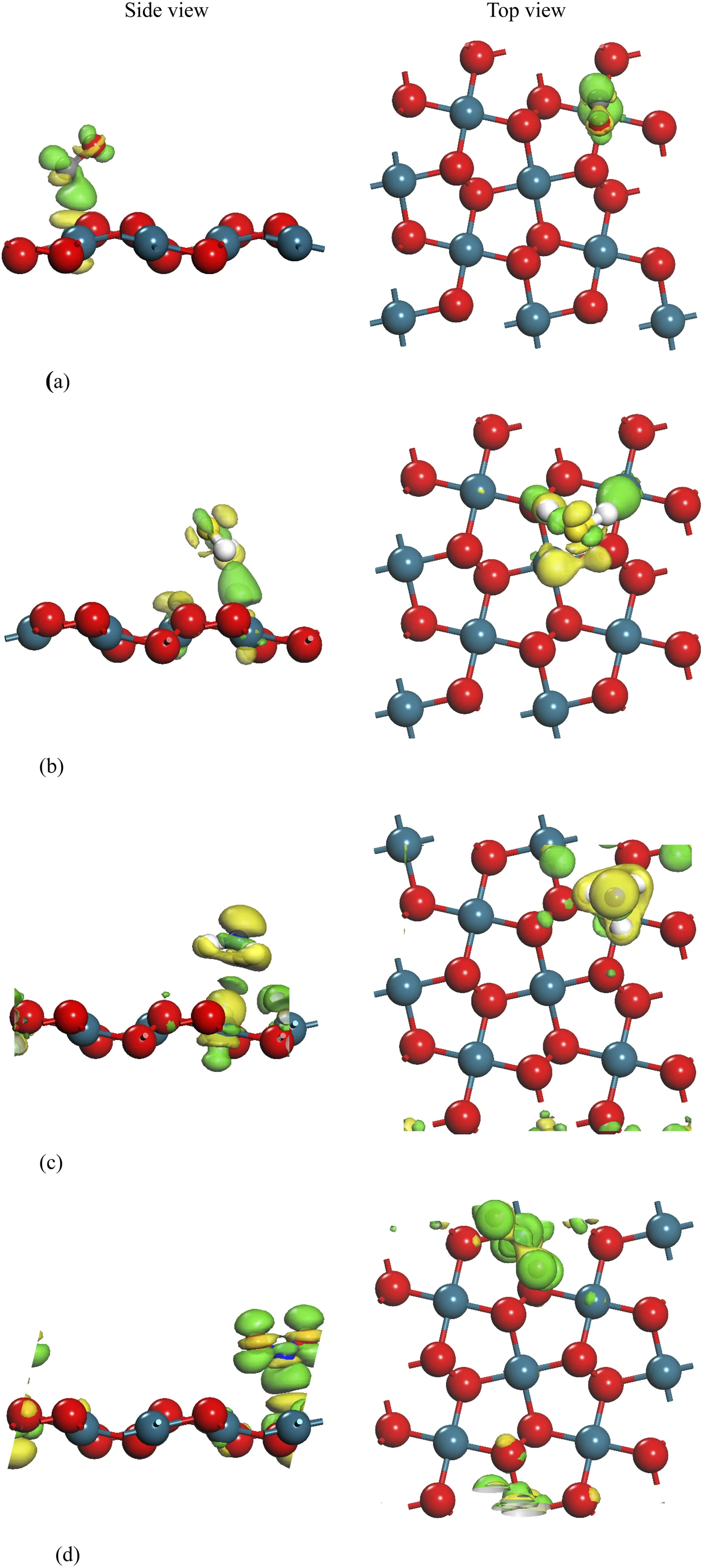
Electron density difference map of complex structures. **(**a) CO+PdO_2_. (b) H_2_S + PdO_2_. (c) NH_3_ + PdO_2_. (d) NO_2_ + PdO_2_.

Fig. 8 displays the electron density difference, EDD, maps used to evaluate charge redistribution and bonding features associated with gas adsorption on the penta-PdO_2_ surface. Electron accumulation appears in the green isosurfaces, whereas depletion is indicated by the yellow regions, revealing how charge redistributes across the surface and helping to interpret the adsorption behavior.

The EDD analysis for CO adsorption on the penta-PdO_2_ surface indicates noticeable charge redistribution upon molecular adsorption, as shown in Fig. 8. Electron depletion is observed around the Pd atom and partially on the C atom of the CO molecule, consistent with their positive Mulliken charges, while accumulation occurs on the O atom and in the Pd–C interfacial region, reflecting localized bonding. These observations suggest that the CO molecule acts as a weak electron donor to the surface.

For H_2_S adsorption, electron depletion is observed on the Pd atom, while accumulation occurs between the H atoms and Pd and on the S atom, consistent with the negative Mulliken charge on S, indicating localized bonding and charge redistribution. The calculated charge transfer confirms that H_2_S donates electrons to the penta-PdO_2_ surface, producing a localized electronic perturbation at the adsorption site. NH_3_ adsorption induces electron depletion on the Pd atom and the H atoms facing the surface, with accumulation between these H atoms and Pd, on the lateral sides of N, and on the surface O atoms, reflecting interfacial bonding and intramolecular charge redistribution. Based on charge transfer, NH_3_ acts as an electron donor, inducing a slight perturbation of the surface electronic structure. The Pd atom shows electron depletion toward NO_2_, while accumulation occurs between Pd and the N atom, indicating interfacial bonding. The lateral sides of N exhibit depletion, and the O atom displays accumulation, consistent with its negative Mulliken charge. By its charge transfer, NO_2_ acts as a strong electron acceptor.

### 3.9 Electron localization function

**Fig. 9:**
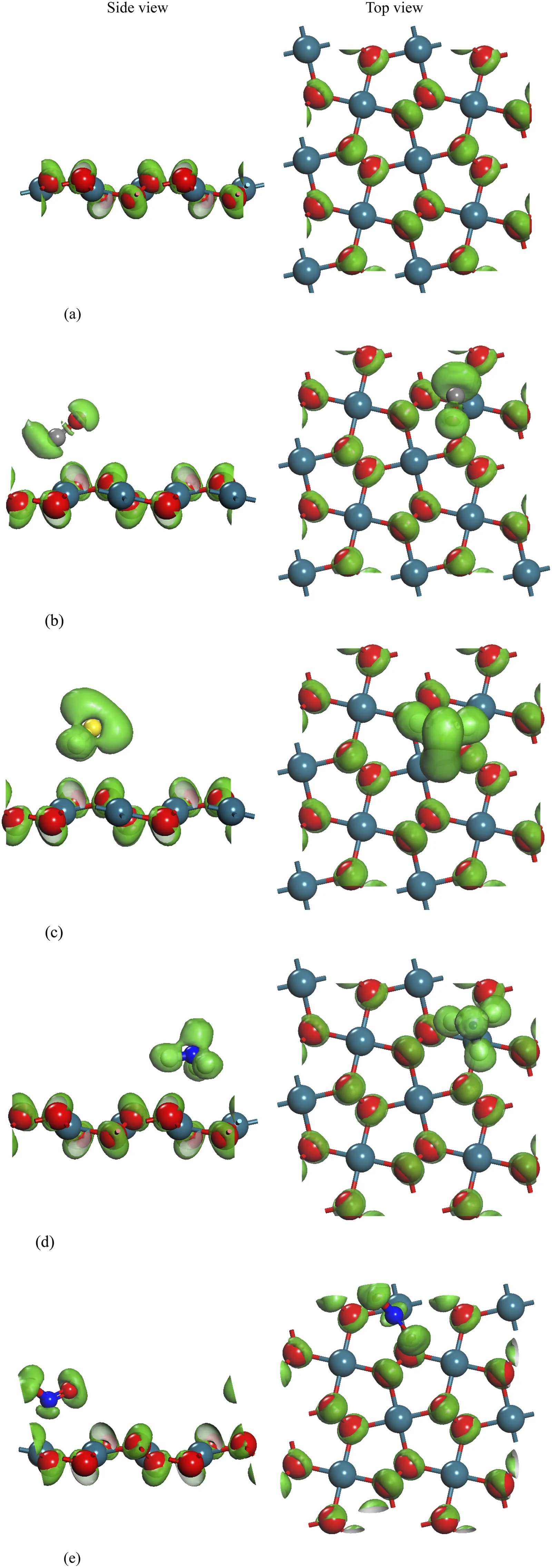
Electron localization function map of pristine and gas-adsorbed structures. (a) PdO_2_. (b) CO + PdO_2_. (c) H_2_S + PdO_2_. (d) NH_3_ + PdO_2_. (e) NO_2_ + PdO_2_.

The spatial distribution of electron pairing in the system is characterized by the Electron Localization Function, ELF.^77^ In the ELF map provided in Fig. 9, regions of high electron localization are marked in green. Because oxygen possesses a higher electronegativity than palladium, the electron density is primarily centered on the O atoms. This distribution underscores the polar character of the Pd–O bonds within the penta-PdO_2_ monolayer. Based on the ELF distributions, the interaction characteristics between the gas molecules and the penta-PdO_2_ surface can be clearly distinguished. The ELF patterns for CO, H_2_S, and NH_3_ show that electron localization is confined within individual species, without the formation of shared regions between the adsorbate and the penta-PdO_2_ surface, indicating weak interaction and physisorption behavior. The ELF map for NO_2_ exhibits a continuous localization region bridging the molecule and the surface, reflecting enhanced interaction and chemisorption.

### 3.10 Electrical conductivity and sensitivity analysis

The sensing response is primarily determined by variations in electrical conductivity upon the adsorption of gas molecules. An increase in the band gap upon gas adsorption reduces conductivity, while a decrease in the band gap enhances it.^78^ Electrical conductivity for pristine and gas-adsorbed configurations is calculated using the following equation:^79^5
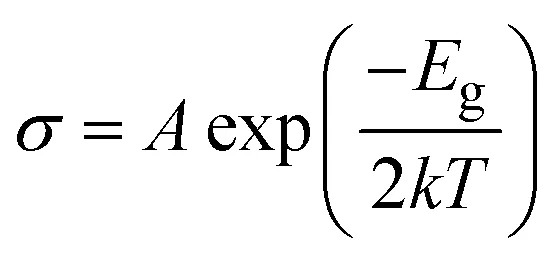
Here, *A* represents the pre-exponential factor, which depends on several transport-related parameters, including carrier mobility, electron–phonon interactions, and scattering mechanisms. Since these parameters cannot be accurately determined for the theoretically predicted penta-PdO_2_ monolayer, this expression is used only to evaluate the relative conductivity variation associated with changes in the band gap rather than absolute electrical conductivity values. Among the investigated gases, NO_2_ adsorption produces the most significant electronic modification by reducing the band gap from 0.855 eV to 0.173 eV, indicating enhanced carrier excitation and conductivity response. CO and NH_3_ adsorption slightly reduce the band gap, suggesting moderate conductivity enhancement, whereas H_2_S adsorption causes a weaker response due to minimal band gap variation. Overall, NO_2_ exerts the most significant influence on penta-PdO_2_, while CO, NH_3_, and H_2_S produce comparatively weaker electronic effects. Gas sensing performance is assessed *via* changes in electrical conductivity induced by gas adsorption, which is commonly used as a measure of sensitivity.^80^ The sensitivity, *S*, of penta-PdO_2_ upon gas adsorption was calculated at 300, 400, and 500 K based on eqn (6), and the corresponding values are reported in Table 3.6
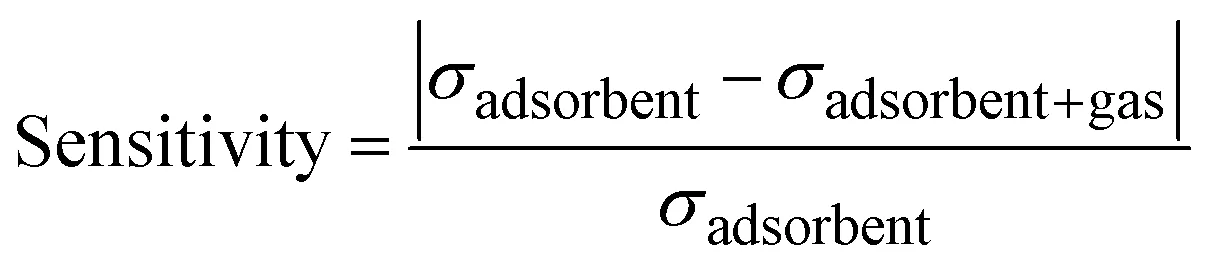
where, *σ*_adsorbent_ and *σ*_adsorbent+gas_ represent the electrical conductivities of the pristine and gas-adsorbed systems, respectively. Among the examined gases, NO_2_ exhibits the highest response, reaching 5.41 × 10^5^ at 300 K, which decreases at higher temperatures, consistent with strong bandgap narrowing and enhanced electrical conductivity upon adsorption. CO and NH_3_ showed moderate sensitivity, peaking at 300 K, while H_2_S displayed very low sensitivity due to minimal bandgap changes and negligible conductivity variation. The consistent decrease in sensitivity with increasing temperature indicates that 300 K is the most favorable operating condition for detection. The overall sensitivity order, NO_2_ > NH_3_ > CO > H_2_S, demonstrates the strong selectivity of penta-PdO_2_ toward NO_2_, primarily arising from changes in the electronic structure and the corresponding increase in electrical conductivity.

**Table 3:** Average Mulliken charge distribution of pristine and gas-adsorbed penta-PdO_2_

Elements	PdO_2_	PdO_2_ + CO	PdO_2_ + H_2_S	PdO_2_ + NH_3_	PdO_2_ + NO_2_
Pd	0.63 e	0.631 e	0.645 e	0.645 e	0.648 e
O	−0.32 e	−0.318 e	−0.316 e	−0.318 e	−0.306 e
C	—	0.33 e	—	—	—
H	—	—	0.09 e	0.323 e	—
S	—	—	−0.28 e	—	—
N	—	—	—	−1.06 e	0.30 e

### 3.11 Work function analysis

The work function, *Φ*, is a fundamental surface property that quantifies the minimum energy needed to move an electron from the Fermi level, *E*_F_, to the vacuum region. It can be expressed as:^81^

7 *Φ* = *V*(∞) − *E*_F_

where *V*(∞) it presents the electrostatic potential in the vacuum far from the material surface. Any variation in *Φ* upon gas adsorption reflects changes in surface charge distribution and the formation of interfacial dipoles, which are critical for sensing behavior. The computed work function values for pristine and gas-adsorbed penta-PdO_2_, Table 4, show a clear dependence on the nature of the adsorbed molecule. The magnitude of work function change follows the sequence: NO_2_ > NH_3_ > H_2_S > CO, indicating progressively stronger electronic interaction with the surface. In the case of CO and H_2_S, only very small shifts in *Φ*, around 0.02 and 0.03 eV, are observed. Such minor variations suggest that these molecules have little influence on the surface potential, which is typical of weak physisorption with negligible charge transfer. A more noticeable change is found for NH_3_ adsorption, where the work function increases by about 0.089 eV. This indicates a moderate level of electronic interaction, likely due to charge donation from NH_3_ to the surface, resulting in a measurable modification of the surface dipole. The largest effect occurs for NO_2_, with a work function shift of approximately 0.12 eV. This substantial increase points to strong coupling between NO_2_ and the penta-PdO_2_ surface, accompanied by significant charge redistribution. This observation is consistent with the marked reduction in band gap and the sharp rise in electrical conductivity reported for this system, confirming that NO_2_ strongly alters the electronic structure. From an application standpoint, these findings are particularly important. Changes in work function provide an alternative sensing signal beyond conventional resistance-based mechanisms. The distinct and molecule-specific *Φ* variations demonstrate that penta-PdO_2_ has strong potential for selective gas detection, with especially high sensitivity toward NO_2_ (Table 5).

**Table 4:** Band gap, (*E*_g_), Fermi energy, (*E*_F_), work function, *Φ*, of the adsorbent and its gas adsorbent complexes

Structures	*E*_g_, (eV)	*E*_F_, (eV)	*Φ*, (eV)	Δ*Φ*, (eV)
PdO_2_	0.855	5.784	5.784	
PdO_2_ + CO	0.826	5.761	5.761	0.023
PdO_2_ + H_2_S	0.861	5.760	5.760	0.024
PdO_2_ + NH_3_	0.825	5.873	5.873	0.089
PdO_2_ + NO_2_	0.173	5.904	5.904	0.12

**Table 5:** Sensitivity of penta-PdO_2_ towards CO, H_2_S, NH_3_ and NO_2_ gas molecules at different temperature

Structures	Sensitivity		
	300 K, (a.u.)	400 K, (a.u.)	500 K, (a.u.)
CO + PdO_2_	0.741	0.525	0.401
H_2_S + PdO_2_	0.111	0.084	0.068
NH_3_ + PdO_2_	0.772	0.547	0.418
NO_2_ + PdO_2_	5.41 × 10^5^	1.97 × 10^4^	2.76 × 10^3^

**Table 6:** Comparison of CO, H_2_S, NH_3_, and NO_2_ gas sensing performance on various 2D nanomaterials based on adsorption energy, (*E*_ads_), band gap, (*E*_g_), charge transfer, (Δ*q*), work function, (*Φ*)*,* and recovery time

Structure	Gas	*E*_ads_ (eV)	*E*_g_ (eV)	Δ*q*, (e)	*Φ* (eV)	Recovery time, (s)	Ref.
PdO_2_	CO	−0.413	0.826	−0.01	5.761	9.6 × 10^−6^	
PdO_2_	H_2_S	−0.378	0.861	−0.10	5.760	2.4 × 10^−6^	
PdO_2_	NH_3_	−0.448	0.825	−0.09	5.873	3.7 × 10^−5^	
PdO_2_	NO_2_	−0.960	0.173	0.71	5.904	1.7 × 10^4^	
MoS_2_	CO	−0.073	—	—	—	—	88
MoS_2_	H_2_S	−0.21	—	0.022	—	—	89
MoS_2_	NH_3_	−0.127	—	—	—	—	88
MoS_2_	NO_2_	−0.138	—	—	—	—	88
TBN	CO	−0.14	3.80	0.00	1.6634	7.70 × 10^−13^	90
InSe	CO	−0.13	1.49	−0.001	—	—	91
InSe	H_2_S	−0.21	1.43	−0.016	—	—	91
InSe	NH_3_	−0.20	1.45	0.019	—	—	91
InSe	NO_2_	−0.24	1.45	0.039	—	—	91
Phosphorene	CO	−0.325	_—_	−0.03	—	—	92
PdSe_2_	NH_3_	−0.287	1.41	−0.029	—	6.61 × 10^−8^	36
PdSe_2_	NO_2_	−0.579	0.52	0.322	—	5.29 × 10^−3^	36
Ti_2_CO_2_	NH_3_	−0.370	—	−0.174	—	—	63
Ti_2_CO_2_	CO	−0.07	—	−0.004	—	—	63
Ti_2_CO_2_	NO_2_	−0.12	—	0.003	—	—	63
B_2_SeTe	CO	−0.017	2.154	0.009	—	1.93 × 10^−12^	93
B_2_SeTe	H_2_S	−0.1	2.166	0.06	—	1.47 × 10^−12^	93
B_2_SeTe	NH_3_	−0.218	2.153	0.053	—	4.56 × 10^−9^	93
B_2_SeTe	NO_2_	−0.528	0.538	0.087	—	7.28 × 10^−4^	93
GaAs	CO	−0.24	1.165	0.0081	5.19	1.03 × 10^−8^	73
GaAs	NH_3_	−0.756	1.15	0.2433	4.842	5.38	73
GaAs	NO_2_	−1.46	1.386	0.2803	5.522	4.45 × 10^12^	73
WSSe	NO_2_	−0.220	—	0.057	—	—	94

In summary, the work function analysis not only supports the interaction strength trend among the studied gases but also highlights the suitability of penta-PdO_2_ as a promising material for work function–based gas sensing technologies.^82^

### 3.12 Optical properties analysis

**Fig. 10:**
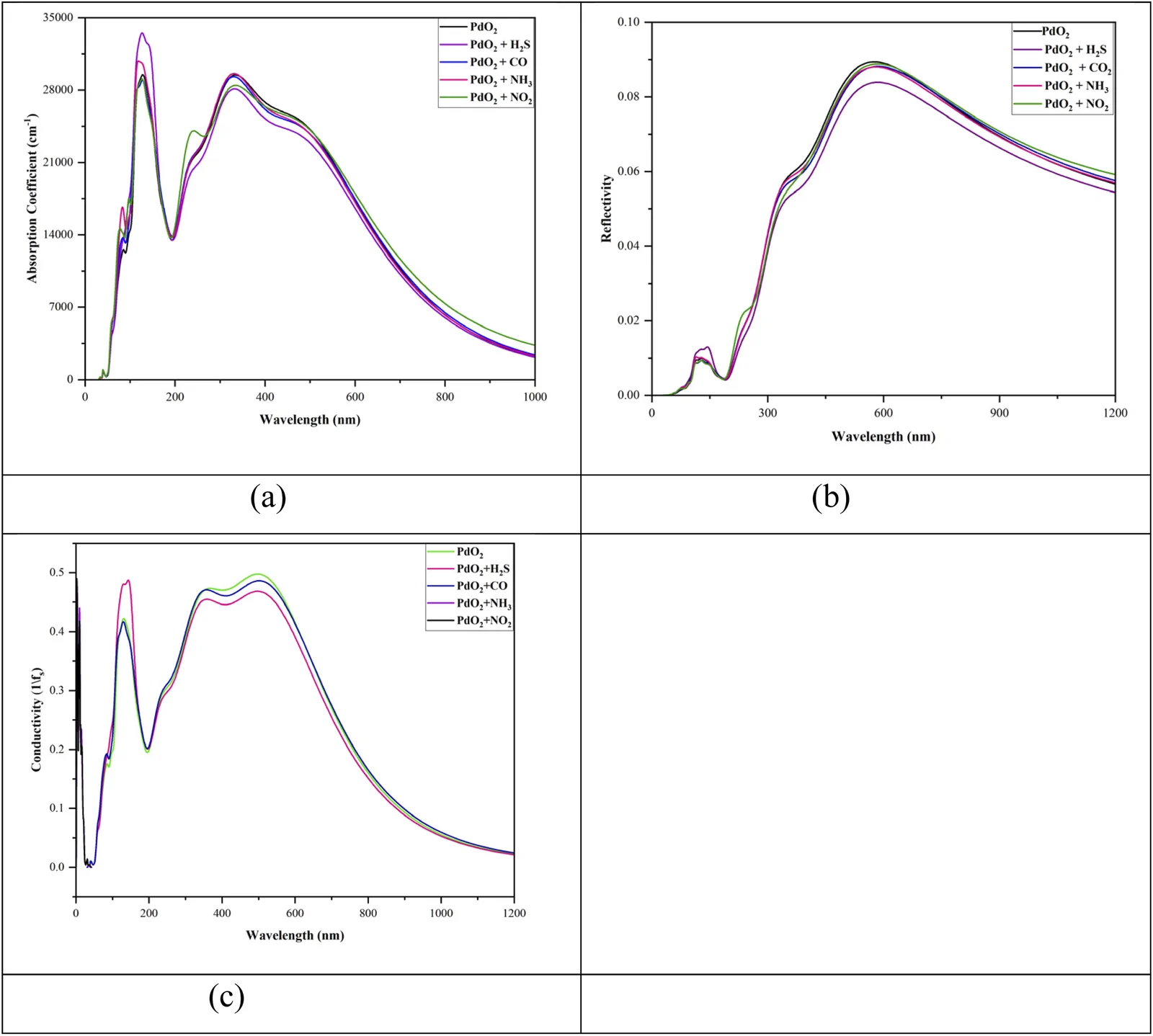
Optical properties analysis for, (a), absorption coefficient spectra of pristine and gas-adsorbed penta-PdO_2_., (b), reflectivity spectra of pristine and gas-adsorbed penta-PdO_2_, (c), optical conductivity spectra of pristine and gas-adsorbed penta-PdO_2_.

The optical behavior of 2D materials is fundamental to their performance in optoelectronic and sensing devices.^83^ To explore how penta-PdO_2_ responds to light, we examined its reflectivity, optical conductivity, and absorption coefficient across both the pristine and gas-exposed surfaces, as shown in Fig. 10. The absorption coefficient spectra for the pristine and gas-adsorbed configurations are presented in Fig. 10a. The pristine monolayer exhibits strong optical absorption in the ultraviolet, UV, region, with a maximum absorption coefficient of approximately 3.1 × 10^4^ cm^−1^ near 150 nm. The absorption intensity gradually decreases toward the visible and near-infrared regions.

Gas adsorption induces noticeable modifications in the optical response of penta-PdO_2_. Among the studied gases, H_2_S slightly enhances the UV absorption intensity, whereas CO and NH_3_ produce only minor changes compared with the pristine system. In contrast, NO_2_ adsorption introduces a distinct absorption feature around 245 nm and maintains comparatively higher absorption in the visible and near-infrared regions, indicating stronger interaction with the penta-PdO_2_.

Distinct variations in the reflectivity spectra are observed for different gas molecules, enabling their effective discrimination.^84^ The reflectivity spectra of penta-PdO_2_ in pristine and gas-adsorbed states are presented in Fig. 10b, showing the distribution of absorbed, transmitted, and reflected photons. The reflectivity spectra reveal that pristine penta-PdO_2_ exhibits a maximum reflectivity of approximately 9.2% near 575 nm. After adsorption, only small variations are observed in the UV region, while H_2_S and NH_3_ induce a slight redshift and reduction in the visible-range reflectivity. CO and NO_2_ adsorption produce comparatively smaller changes.

The conductivity spectra of penta-PdO_2_ in the pristine and adsorbed states are presented in Fig. 10c. All systems exhibit high conductivity in the UV region, <400 nm, H_2_S adsorption slightly enhances the conductivity near 140 nm, whereas NO_2_ causes a small redshift in the visible region without significant intensity variation. Overall, the optical analysis indicates that gas adsorption modulates the optical response of penta-PdO_2_, with NO_2_ producing the most pronounced effect due to its strong electronic interaction with the surface.

Overall, the optical analysis indicates that gas adsorption modulates the optical response of penta-PdO_2_ to varying degrees. CO, H_2_S, and NH_3_ induce relatively small changes, whereas NO_2_ causes more pronounced modifications due to stronger interaction with the surface. These results demonstrate the sensitivity of penta-PdO_2_ toward different gas molecules and highlight its potential for optical gas-sensing applications.

### 3.13 Reduced density gradient, RDG, analysis

**Fig. 11:**
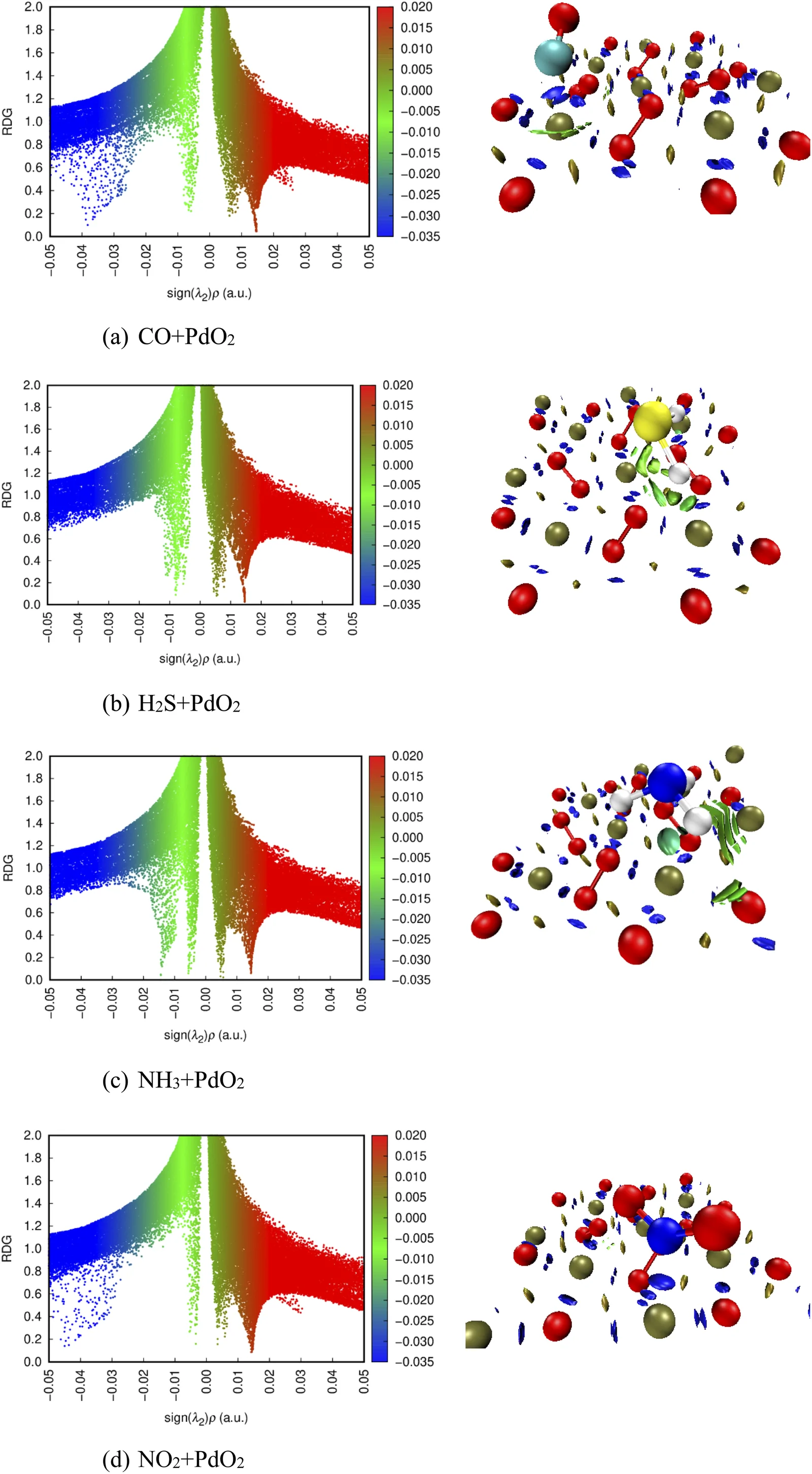
Illustration of RDG isosurfaces and corresponding scatter plots for, (a), CO + PdO_2_, (b), H_2_S + PdO_2_, (c), NH_3_ + PdO_2_, and, (d), NO_2_ + PdO_2_.

Non-covalent interaction, NCI, analysis is a computational approach used to investigate weak intermolecular forces present in molecular systems. Such interactions include hydrogen bonding, van der Waals attractions, and dipole–dipole forces, all of which arise from electrostatic or transient charge distributions rather than electron sharing. Although these interactions are significantly weaker than covalent bonds, they have a major influence on the organization, stability, and overall behavior of molecular assemblies.^85^ The Reduced Density Gradient, RDG, method serves as the primary computational tool in NCI analysis, enabling the localization and visualization of regions where these weak interactions occur, thus offering detailed insights into molecular behavior and interaction patterns.^86^ It produces a two-dimensional RDG scatter plot along with a three-dimensional gradient isosurface, enabling detailed visualization of non-covalent interaction regions, their relative intensities, and spatial distribution within the system. The RDG scatter plots and their corresponding gradient isosurfaces for the gas-adsorbed complexes are presented in Fig. 11, computed using the equation below :^85^8
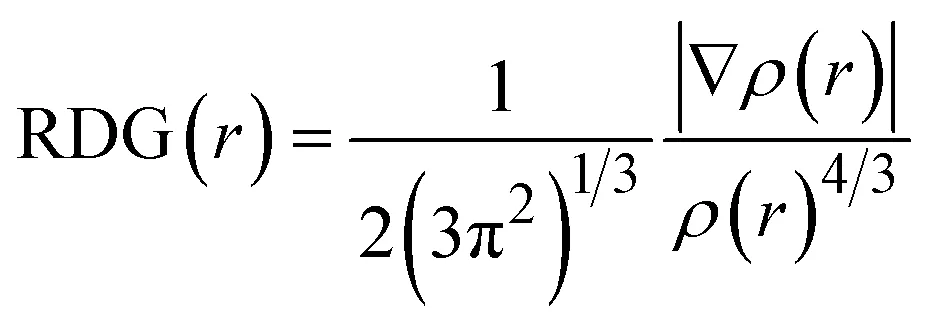
Here, the analysis of the electronic environment is carried out in real space by considering the electron density *ρ*(*r*) along with its spatial variation described by ∇*ρ*(*r*). The reduced density gradient, RDG, analysis is based on the electron density *ρ*(*r*) and its spatial derivatives. The nature of the interaction regions is further characterized using the sign of *λ*_2_(*r*), where *λ*_2_(*r*) corresponds to the second eigenvalue of the electron density Hessian matrix. This descriptor enables the classification of different interaction regimes in real space. Regions with negative values are associated with attractive interactions, whereas positive values correspond to steric repulsion. In contrast, values approaching zero are indicative of weak dispersion, van der Waals, interactions. The interaction regions are visualized through color-coded isosurfaces obtained from Hessian analysis. Blue regions correspond to attractive electrostatic interactions, whereas red indicates steric repulsion. Weak van der Waals interactions are represented by green isosurfaces.^87^

The RDG analysis reveals distinct interaction characteristics between the gas molecules and the penta-PdO_2_ surface. For NO_2_ adsorption, the prominent blue regions observed in both the isosurface and scatter plot indicate strong attractive interactions associated with electrostatic coupling and partial orbital hybridization, confirming chemisorption behavior. In contrast, CO, H_2_S, and NH_3_ adsorption mainly exhibit green isosurfaces with weak features near sign, *λ*_2_, *ρ* ≈ 0, suggesting that their adsorption is dominated by weak van der Waals interactions.

Overall, the RDG results indicate that NO_2_ interacts most strongly with the penta-PdO_2_ monolayer, whereas CO, H_2_S, and NH_3_ are primarily governed by weak noncovalent interactions.

## 4. Conclusion

In this work, a novel penta-PdO_2_ monolayer was systematically investigated as a potential two-dimensional gas-sensing material using DFT calculations. The calculated cohesive energy and phonon dispersion suggest the energetic and dynamical stability of the monolayer, indicating that the proposed structure could be suitable for gas-sensing applications. Pristine penta-PdO_2_ exhibits intrinsic semiconducting behavior with an indirect band gap, providing a favorable platform for adsorption-induced electronic modulation. The adsorption analysis revealed distinct sensing characteristics for different gas molecules. CO, H_2_S, and NH_3_ interact weakly with the surface through physisorption, resulting in small charge transfer, minimal structural distortion, and rapid recovery behavior, which may be advantageous for reversible sensing operation. In contrast, NO_2_ exhibits comparatively strong chemisorption with large adsorption energy and significant charge redistribution, leading to pronounced orbital hybridization, enhanced electronic coupling, notable band-gap reduction, and spin-polarized magnetic behavior, highlighting the potential sensitivity and selectivity of penta-PdO_2_ toward NO_2_ detection. Further analyses, including density of states, electron density difference, electron localization function, and RDG analysis, consistently indicate weak noncovalent interactions for CO, H_2_S, and NH_3_, whereas NO_2_ adsorption strongly influences the electronic structure of the monolayer. In addition, NO_2_ adsorption is predicted to significantly enhance the electrical conductivity and modulate the work function, resulting in a comparatively higher sensing response than the other investigated gases. Optical analysis also suggests noticeable changes in the absorption and conductivity spectra, particularly after NO_2_ adsorption, further supporting its strong interaction with the monolayer. Although the present study focuses on the intrinsic properties of the free-standing penta-PdO_2_ monolayer, practical gas sensors will require integration with suitable substrates and metallic electrodes. These interfaces may influence the adsorption behavior and electronic response through interfacial interactions. Future studies incorporating realistic device architectures and Boltzmann transport calculations will further bridge the gap between theoretical predictions and experimental implementation. Overall, these findings indicate that penta-PdO_2_ could serve as a promising candidate for selective NO_2_ adsorption and toxic gas sensing applications.

## Author contributions

Methila Akter: formal analysis, data curation, investigation, visualization, writing – original draft, Abu Talha: formal analysis, investigation, visualization, writing – original draft, Nuzhat Nawshin: formal analysis, visualization, writing – original draft, Mohammad Tanvir Ahmed: resources, software, validation, writing – review & editing, Debashis Roy: formal analysis, project administration, validation, supervision, writing – review & editing, Abdullah Al Roman: conceptualization, methodology, project administration, supervision, writing – review & editing.

## Conflicts of interest

The authors of this manuscript have no conflicts of interest to declare.

## Data Availability

Data associated with this manuscript will be shared upon reasonable request.
